# Promoting Child Wellness: A Narrative Review of Positive Childhood Experiences

**DOI:** 10.3390/bs15111432

**Published:** 2025-10-22

**Authors:** Cynthia R. Rovnaghi, Donovan Castilla-Liu, Ashley M. Lee, Akul Shrivastava, Kanwaljeet J. S. Anand

**Affiliations:** 1 Child Wellness Lab, Stanford University School of Medicine, Palo Alto, CA 94305, USAanandam@stanford.edu (K.J.S.A.); 2 Department of Pediatrics, Anesthesiology & Pain Medicine, Stanford University School of Medicine, Palo Alto, CA 94305, USA

**Keywords:** adverse childhood experiences, positive childhood experiences, well-being, flourishing

## Abstract

Positive childhood experiences (PCEs) are increasingly recognized as critical factors that promote resilience, emotional regulation, and flourishing in children, especially in the context of adversity. This narrative review explores the conceptual development, empirical evidence, and theoretical frameworks underpinning the role of PCEs in early childhood development. A critical assessment of the existing literature focuses on how PCEs function as promotive and protective factors and evaluates the strengths and limitations of current measurement tools. Drawing on theories from resilience science, developmental psychopathology, positive psychology, and ecological systems theory, this review highlights the complex, multidimensional nature of PCEs and their interplay with parenting styles, socioeconomic status, and the social drivers of health. Despite compelling evidence that PCEs influence cognitive, emotional, relational, and behavioral outcomes across the lifespan, there is no dedicated validated tool for prospectively measuring PCEs in preverbal or preschool-aged children. This gap limits our ability to design and test interventions to mitigate adverse childhood experiences and to assess their developmental impact in real time and subsequent periods. We conclude that future research should focus on creating culturally sensitive, developmentally appropriate instruments to measure PCEs in early life, essential for advancing equity, optimizing child health, and promoting wellness across diverse populations.

## 1. Introduction

A baby’s birth brings a lot of joy and excitement to families, forever changing the family dynamics. Parents, siblings, grandparents, neighbors, and others are enamored by this new life, doing their utmost to create a nurturing, comforting, and supportive environment for their child. Not only are the immediate well-wishers, but entire communities, societal bodies, governments, and nations are designed, to some extent, to provide the structures and resources most conducive for all children to thrive (C. D. Bethell et al., 2019; CDPH, 2023). Some children, however, may not be born into circumstances as favorable as those created by a nurturing family and community, often exposing them to deprivation, adversity, insecurity, neglect, or abuse. Other children may be born with or acquire health-related concerns that disrupt their normal growth and development at different stages of their childhood.

Families and communities often use multiple approaches to mitigate a child’s outcomes associated with health-related concerns that disrupt growth and development, or upon exposure to deprivation, adversity, insecurity, neglect, or abuse. Within the physical, economic, and social ecology of early childhood (Lopez et al., 2021), we discuss the importance of positive childhood experiences (PCEs) in promoting child well-being, resilience, and flourishing (Crouch et al., 2023; Huppert & So, 2013). PCEs are conducive, favorable factors that contribute to building resilience, sustaining all domains of health, and promoting child flourishing. Resilience is the ability to successfully navigate, adjust to, or cope with major stressors or traumatic experiences (Windle, 2011). Flourishing is distinguished as the implementation of positive, adaptive coping mechanisms to achieve emotional regulation, cognition, and executive function in fortifying health (Huppert & So, 2013). Well-being, a component of flourishing, is the state of sustained physical and emotional health (Huppert & So, 2013). Ample research in adolescence and adulthood demonstrates that the physical and psychosocial maturational stages are linked with genetic–epigenetic components responsible for guiding brain development, executive function, cognitive capacity, and brain/behavior mechanisms (Lossi et al., 2024; Miguel et al., 2023; Ng et al., 2023). Despite their critical role in early brain development, PCEs have not been systematically explored in preschool-aged children for their ability to reverse allostasis-associated dysregulation of the brain–mind–body connectome. We explore prevailing theories and frameworks shaping our understanding of PCEs alongside the evolution of measurement tools aimed at capturing their multifaceted nature. This narrative review aims to critically assess existing research on PCEs, while offering insights into the future avenues for advancing our comprehension of early child development.

## 2. Overview of Positive Childhood Experiences

### 2.1. Defining PCEs

A PCE is an event, interaction, or circumstance that beneficially contributes to a child’s resilience and thus to their development, well-being, and healthy long-term outcomes. PCEs include supportive, stable relationships, safe and nurturing environments, cultural and spiritual connections, as well as opportunities for equitable education and health care access, among others. Broadly speaking, PCEs can facilitate and maintain healthy development by stabilizing emotional regulation with cognitive processing shaped by the practice of resilience skills, namely, learned positive coping strategies (C. Bethell et al., 2019; Crandall et al., 2019; Hanson et al., 2024; Yu et al., 2022). Research suggests that PCEs embody a stress exposure-resilience building response relationship, signifying that emotional-cognitive stability is dependent on the sufficient aggregation of PCEs during childhood (C. Bethell et al., 2019).

Much of the current PCE literature concerns multidimensional relationships, the daily environment, and the internal psyche of school-aged children. School-aged children cultivate relationships on the individual, familial, and communal levels. These can include healthy, supportive relationships with parent(s), peers at school, teachers, mentors, and other members of the community (Racine et al., 2020). Others emphasize the environment of the child, such as having a predictable daily routine or a safe, stable, protective, and learning environment (Han et al., 2023; R. D. Sege & Harper Browne, 2017). Yet others focus on child psychology: the development of self-esteem or positive core beliefs (R. D. Sege & Harper Browne, 2017). Together, these foci on positive relationships, environmental context, and child psychology are key, though should not be limiting, when categorizing PCEs.

Implicit in these criteria is access to resources that enable children to lead healthy lives. Unfortunately, many children do not have adequate access to what the Centers for Disease Control and Prevention (CDC) terms social determinants of health (SDoH), namely, economic stability, high-quality educational and healthcare access, secure neighborhood and home environments, and contextual networking with social community support (CDC, 2024b). We prefer to term these factors as the Social *Drivers* of Health (SDH), recognizing that these factors do not *determine* our health status, but rather influence it in multiple ways. Currently, many children in lower socioeconomic status (SES) families are less likely to experience PCEs during their childhood (R. Sege et al., 2024). People of color were more likely to score higher on the socioeconomic adversity index (SAI) and thus experience outcomes like people of lower SES (Anand et al., 2019). Furthermore, both adverse childhood experiences (ACEs) and PCEs can be transmitted vertically across generations, exacerbating this inequality (Narayan et al., 2021). Less is known about PCEs and ACEs in the context of culturally based practices in child rearing. Child rearing, disciplinary, educational, religious, work, and health practices may vary culturally, and acculturation may contribute to inequity with significant acculturation stress (Agorastos et al., 2019; Al-Jayyousi et al., 2021; Pittaway et al., 2023; Shaligram et al., 2022).

Children growing in apathetic or hostile environments may suffer from stifled development or receptivity to PCEs. The social domain perspective hinges on a child’s emotional and cognitive adaptive skills that are intertwined with a sense of belonging rooted in the social, conventional, moral, and personal spheres of influence. The moral domain concerns issues of welfare and rights, the social conventional domain directs traditions and social norms, and the personal domain describes actions that are outside the bounds of moral or social conventions, such as personal preferences (Smetana et al., 2014). By viewing a child’s actions through these domains, their family, community, or peers may choose to address the child’s behavior in unhealthy ways. For instance, in hostile environments, actions that could be positive for childhood development may be seen as falling in the social conventional or moral domain, and thus a family or community may seek to forcefully curb such behavior, stifling a child’s development and sense of cooperation. In an apathetic environment, the community may view actions (parental and/or child) detrimental to a child’s health and development as falling into the personal domain of parental prerogatives, thus allowing the problematic parent–child behaviors to persist.

This review elaborates on the roles of PCEs in mediating resilience-building skills supportive of emotional regulation, cognitive development, executive function, and good health. Whereas much of the past literature focused exclusively on the buffering effects against adversity and promotion of resilience, current publications seek to clarify how PCEs serve essential roles in facilitating normal childhood development. A developmental systems framework adds that an individual’s resilience is affected by personal capabilities that are shaped by their relationships with others (Masten, 2019). Many investigations have studied PCEs in terms of protective or promotive resilience factors. We follow Han et al. (2023) in distinguishing these two factor types: A promotive PCE shows a main effect improving outcomes across risk strata (C. Bethell et al., 2019), and a protective PCE buffers the negative effect of adversity, appearing as an interaction such that the factor reduces the ACEs→outcome slope. Some PCEs may serve a dual role, such as secure attachment, which shows both direct promotive associations with socioemotional competence and the buffering of physiologic stress responses under adversity (Han et al., 2023; Morris et al., 2016).

### 2.2. Empirical Evidence for PCEs

Of note, PCEs may serve as promotive factors during child development and may become protective during exposures to adversity (Han et al., 2023). Developmental psychopathologists define promotive PCEs as factors associated with favorable childhood outcomes in low- and high-risk contexts (Masten & Cicchetti, 2016; Narayan et al., 2018). Conversely, protective factors are traditionally defined as moderators or buffers capable of reducing the probability of unfavorable outcomes in the face of increasing adversity and raising the probability of positive adaptation (Narayan et al., 2018; Wright et al., 2013).

Protective PCEs can counteract the negative developmental effects of adversity. Most literature emphasizes the moderation of ACEs through learned resilience skills mediated by PCEs (C. D. Bethell et al., 2014; Masten, 2019; Narayan et al., 2021). A meta-analysis revealed that self-regulation supported by the family, peers, and school staff was consistent in fostering resilience in the children exposed to violence (Yule et al., 2019). These personal and environmental factors likely promote resilience in other contexts of adversity or trauma as well. For example, higher mastery motivation, namely, the drive to control and master challenges, was associated with better social and emotional functioning and emotion regulation in young children facing homelessness (Ramakrishnan & Masten, 2020).

The familial support aspect is a prominent focus of investigation. For instance, secure attachments between infants and mothers observed at 18 months predicted smaller amygdala volume in early adulthood (Moutsiana et al., 2015). We envision PCEs as opportunities for parent, child, family, and community engagement that motivate a child toward optimal growth and lifestyle. Child motivation with hope and positive health are dependent on active participation (play, listening, setting goals); through establishment and practice of clear guidelines, expectations, routines, independent (self-regulated) and cooperative behaviors; and positive reinforcements of effort and progress (Center on the Developing Child at Harvard University, 2018). Thus, familial resilience and connections can increase child coping mechanisms by inculcating child self-regulation toward positive outcomes in response to mental, emotional, or behavioral problems (C. Bethell et al., 2019; C. D. Bethell et al., 2022). The quality of parenting can buffer against a child internalizing problems (Labella et al., 2019). Homeless parents who exhibit fewer maladaptive cognitive–emotional regulation strategies and more effective parenting reduced their young child’s risks of internalizing symptoms at school (Palmer et al., 2020).

Supportive parenting was associated with maintained resting-state functional connectivity (rsFC) in both the central-executive network and an emotion-regulation network during early adulthood, which reduced the effects of adolescent poverty (Brody et al., 2019). Among adolescents exposed to poverty, caregiver support was associated with dorsal frontal lobe and amygdala development similar to that of children with lower levels of socioeconomic disadvantage (Whittle et al., 2017). In an experimental social-exclusion task, individuals with higher social support showed lower cortisol reactivity and decreased dorsal anterior cingulate cortex (dACC) activity; lower dACC activity in that study was associated with lower perceived stress (Eisenberger et al., 2007).

Taken together, these findings suggest a possible mechanistic pathway by which social support contributes to resilience: social support may reduce dACC activity, thereby attenuating stress responses (a mechanism discussed in reviews such as Ditzen & Heinrichs, 2014). However, this mechanism remains interpretive; while some experimental and correlational data are consistent with it, causal mediation across different contexts has not been fully established. Finally, it is important to note that protective caregiving experiences (PCEs) likely do not offset all risks and may not confer equal protection across differing profiles of adverse childhood experiences (ACEs), early life stress (ELS), or traumatic events; the extent and boundary conditions of PCE protection require further empirical specification.

PCEs can serve as promotive factors supporting adaptation with resilience-building skills affecting the mental, physical, and social aspects of a child’s flourishing health. For instance, PCEs predict decreased chances of poor mental health, such as depressive or anxiety disorders (C. Bethell et al., 2019; Kuhar & Zager Kocjan, 2021). Parents who engage in singing and storytelling with children below age five also promote child flourishing, enhancing their ability to cope with stressful situations (Waters et al., 2023). PCEs may also predict better cardiovascular health, more physical exercise, and less cigarette use across the lifespan, promoting better health outcomes (Graupensperger et al., 2023; Kosterman et al., 2011; Slopen et al., 2017). However, the accuracy of these associations with physical health may be improved by accounting for childhood adversity (Han et al., 2023).

Strong evidence linking the interplay between parenting styles and childhood outcomes shows that parenting styles can regulate a child’s brain and behavioral development and the child’s health (Tohi et al., 2022), nutritional status (Islam et al., 2024; Li et al., 2024; Sanchez-Martinez et al., 2024) and sleep habits (Brand et al., 2009; Gorgoni et al., 2013; Urbain et al., 2014). Certain parenting styles promote a child’s ability to draw upon positive experiences and develop coping strategies that reduce internalizing and externalizing behavioral problems (Ciriegio et al., 2025). For example, cooperation, a prosocial behavior, is supported by parent–child secure bond formation with brain synchrony observed in the dorsolateral prefrontal cortex (DLPFC) and frontopolar cortex (FPC) (Reindl et al., 2018). Oxytocin release in infants in response to caregiver support facilitates the development of trust and additional prosocial behaviors (Meyer-Lindenberg et al., 2011; Smith & Wang, 2012). PCEs are correlated with enhanced prosocial behaviors and reduced aggressiveness (Narayan et al., 2023; Zhan et al., 2021).

Children’s anticipatory behaviors, such as looking forward to routines, engaging in imaginative play, and expressing excitement for future events, are also strongly linked to PCEs. These behaviors indicate a sense of security and emotional well-being, fostered by supportive relationships, stable environments, and opportunities for exploration (C. Bethell et al., 2019; C. D. Bethell et al., 2019). Research suggests that children who experience consistent positive interactions with caregivers and a nurturing community develop stronger coping mechanisms and resilience, allowing them to approach new situations with confidence and optimism (R. D. Sege & Harper Browne, 2017). By encouraging anticipatory behaviors through structured yet flexible environments, caregivers and educators can help children build trust in their surroundings, reinforcing a foundation for healthy emotional and cognitive development.

Preferential looking, a measure of visual attention in infants, is a key indicator of early cognitive and emotional development. While direct studies linking preferential looking to PCEs are limited, research suggests that nurturing and responsive caregiving environments can significantly influence infants’ attentional patterns. For instance, one study found that infants who looked longer at their mother’s face at 6 months exhibited a better ability to recover from distress at 9 months, suggesting a link between early visual attention and emotional regulation (Rigato et al., 2023).

Although multiple studies report links between positive childhood experiences (PCEs) and improved mental and physical health, important limitations temper causal inference. Most large-scale syntheses and primary studies rely on cross-sectional or retrospective designs that are vulnerable to recall bias and confounding by socioeconomic status, parental mental health, and concurrent family resources (Han et al., 2023). Neuroimaging and biomarker studies vary widely in sample size, age range, and analytic approach, reducing comparability and leading to heterogeneous effect sizes (Gee et al., 2013; Gunnar, 2002). Taken together, the extant literature robustly supports associations between aggregated PCEs and later outcomes, but definitive evidence for causal mechanisms, particularly in infancy and toddlerhood, requires prospective, repeated-measures designs, preregistered analytic plans, and consistent operational definitions across studies (Han et al., 2023; Narayan et al., 2023).

### 2.3. Parental Adversity, PTSD, and PCEs in the Context of Parental-Child Relationships

Almost all publications centered on outcomes related to adversity or trauma and PCEs collectively focus on individuals at or above school age. Less is known about how PCEs shape a young child’s sense of value and self-worth. A validated instrument for assessing PCEs in preschool children does not exist. Any survey of PCEs in preschool children must minimally assess parenting styles, the quality of social relationships (parent–child, family, friends, community), and the influence of parents’ own experiences of stress and support with or without their own experiences of early life adversity/trauma. Collectively, these parental experiences shape a child’s sense of security and value, social-emotional development, problem-solving skills, growth, executive function, and health outcomes. We previously found that marital status, parental education, annual household income, and household status moderate a child’s cognitive and emotional outcomes (Anand et al., 2019). Most recently, McDorman et al. (2025) found that parenting relationships were more strongly related to children’s executive functions and social-emotional skills than either low SES or high household chaos, indicating the importance of parenting relationships for children’s social-emotional development across different risk levels (McDorman et al., 2025). High levels of parenting stress are associated with an increased likelihood of children experiencing ACEs and a decreased likelihood of experiencing PCEs within the home, school, and community (Crouch et al., 2019, 2024).

Parents’ ability to surmount their own stress, to instill a young child’s acceptance of cooperation, and encouragement of the child’s positive self-regulation from anticipated and predictable daily routines for eating, sleeping, playing, learning, and bathing are crucial to maintain a child’s physical and mental health. Rahim et al. (2025) elaborate on the complex relationships between circadian rhythms and psychological health, suggesting that mood dysregulation may arise from systemic misalignments between external routines and internal synchronization of circadian clocks (Rahim et al., 2025). Pediatricians worldwide commonly rely on height/weight measurements to provide critical insights into a child’s physical growth, enabling the identification of growth abnormalities or nutritional deficiencies (Alimujiang et al., 2018). In contrast, sleep quality serves as a window into a child’s mental and emotional well-being. Sleep-dependent brain plasticity allows young brains to reorganize, grow, and refine neuronal networks for learning and memory (Gorgoni et al., 2013; Urbain et al., 2014). High-quality sleep is essential for cognitive development, emotional regulation, and behavioral health, as well as physical growth via the release of growth hormones during deep sleep (Schlieber & Han, 2021). While height and weight primarily address physical aspects of development, sleep quality bridges the physical and mental domains, highlighting its dual role in fostering both bodily health and psychological resilience. This underscores the importance of integrating sleep assessments into pediatric care along with traditional growth metrics. Given the critical role of sleep in both physical and mental health, it is essential to consider the broader influences on sleep quality, particularly the impact of parenting styles.

Parenting styles have long been recognized as a critical factor in child development, influencing various aspects of children’s lives, including their academic achievement, social competence, and psychological well-being. The seminal work of Baumrind and other researchers has consistently demonstrated that different parenting styles are associated with distinct developmental outcomes in youth (Kuppens & Ceulemans, 2019). The literature typically identifies four main parenting styles: authoritative, authoritarian, permissive, and neglectful. These styles are characterized by varying levels of responsiveness and demandingness, which significantly influence child sleep and wellness (Smetana, 2017; Yang et al., 2020). Authoritative parenting, marked by high responsiveness and demandingness, is often considered the ideal approach. Authoritative parenting was consistently associated with better sleep outcomes in young children, consistent bedtime routines, responsive caregiving, secure attachment and healthy sleep patterns. In contrast, authoritarian parenting, characterized by high demandingness and low responsiveness, can potentially hinder the development of secure attachment; while potentially effective in enforcing sleep schedules, it may create anxiety that disrupts sleep quality. Permissive parenting, characterized by high responsiveness but low demandingness, can foster strong emotional bonds, but it may also result in inconsistent sleep patterns due to a lack of structure (Tyler et al., 2019; Yang et al., 2020). Neglectful parenting, with low responsiveness and low demandingness, is likely to have the most negative impact on bonding, sleep quality, and child wellness. Children raised with this style may struggle with emotional regulation and sleep disorders due to a lack of guidance and support (Kuppens & Ceulemans, 2019).

As a child enters adolescence, the relationship between parenting styles and sleep wellness evolves, but the patterns established in early childhood often persist. *Authoritative parenting* continues to be associated with better sleep outcomes, as it provides a balance of structure and autonomy that aligns well with adolescent developmental needs. *Authoritarian parenting* may face increased resistance from adolescents, potentially exacerbating sleep issues (Baumrind et al., 2010; Zapata Roblyer & Grzywacz, 2015). *Permissive parenting* might struggle to instill healthy sleep habits in the face of increasing external influences, while *neglectful parenting* leads to significant sleep problems as adolescents lack the necessary guidance to navigate this developmental stage (Baumrind et al., 2010).

The relationship between parenting styles and parental self-esteem is bidirectional and complex. Parents who adopt an authoritative style often report higher levels of self-esteem. This suggests that parents who feel more confident and have a positive self-image are better equipped to provide warmth, support, and create boundaries for their children. The relationships between other parenting styles and parental self-esteem are still unclear, with parental education, socioeconomic status, and mental health acting as confounding variables (Aunola et al., 1999).

Authoritative parenting consistently emerges as the most beneficial style for promoting secure attachment and healthy sleep habits across developmental stages, while neglectful parenting presents the most risks (Baumrind et al., 2010; Kuppens & Ceulemans, 2019). Authoritarian and permissive styles occupy intermediate positions, each with distinct advantages and challenges (Tyler et al., 2019). The adoption of effective parenting styles appears to be positively correlated with parental self-esteem, highlighting the reciprocal nature of parent–child relationships (Aunola et al., 1999). Interventions aimed at promoting authoritative parenting practices may prove beneficial for enhancing both child outcomes and parental self-esteem across all stages of development by increasing opportunities for child–parent PCEs and decreasing opportunities for child–parent ACEs.

### 2.4. Positive Childhood Experiences in Practice

Parenting practices, particularly in early childhood, play an important role in exposing children to PCEs. The frequency and variety of PCEs hinge on many elements of the parent–child relationship. Researchers have begun to explore how PCEs associated with parent–child attachment, positive parenting (e.g., parental warmth and support), and family health can support childhood development while mitigating the potential detrimental or damaging consequences of exposure to ACEs (C. Bethell et al., 2019). Secure attachment, for example, significantly improves the outcomes for children across a range of domains, including social, emotional, behavioral, and relational health, along with academic achievement (Barlow et al., 2015, 2016; Dalgaard et al., 2022). Researchers have recently explored the role of positive parenting in fostering child wellness, defined by Seay et al. (2014) as “*…the continual relationship of a parent(s) and a child or children that includes caring, teaching, leading, communicating, and providing for the needs of a child consistently and unconditionally*.”

The promotion of positive parenting practices is crucial to creating the environments necessary for children to flourish and grow, whereas a lack of positive parenting increases the risks of social, emotional, or behavioral problems later in life (Barlow & Coren, 2018; Seay et al., 2014). While exploring the potential mitigating effect of PCEs on the adverse consequences of ACE exposure, research has shown dose–response associations of PCEs with future health outcomes, protecting youth from risky sex, substance abuse, depression, and promoting a more positive body image (Crandall et al., 2020), while preventing mental health problems in adolescents and adults (C. Bethell et al., 2019; Qu et al., 2022). In other words, PCEs promoted through positive parenting practices and family dynamics can moderate the impact of ACEs on future mental and physical health outcomes, underscoring the importance of early and proactive promotion of PCEs in young children.

To promote PCEs in early childhood, researchers have investigated various types of parenting programs that effectively teach positive parenting practices. Several studies reveal that both group- and individual-based parent training, including parent–child psychotherapy, mentalism, video-feedback training, and mindfulness-enhanced parenting programs, have the potential to promote secure attachment, improve parenting skills, and reduce harsh parenting practices (Barlow et al., 2015, 2016; Barlow & Coren, 2018; Dalgaard et al., 2022; Featherston et al., 2024). Typically, parenting programs last between 8 and 12 weeks with a frequency of 1–2 h each week, with outcomes being measured pre- and post-intervention (Barlow & Coren, 2018). Further research is needed to better understand the long-term effects of parental training on the promotion of positive parenting practices and PCEs, as well as the overall health and well-being of the child.

## 3. Theories & Frameworks Related to PCEs

**Figure 1:**
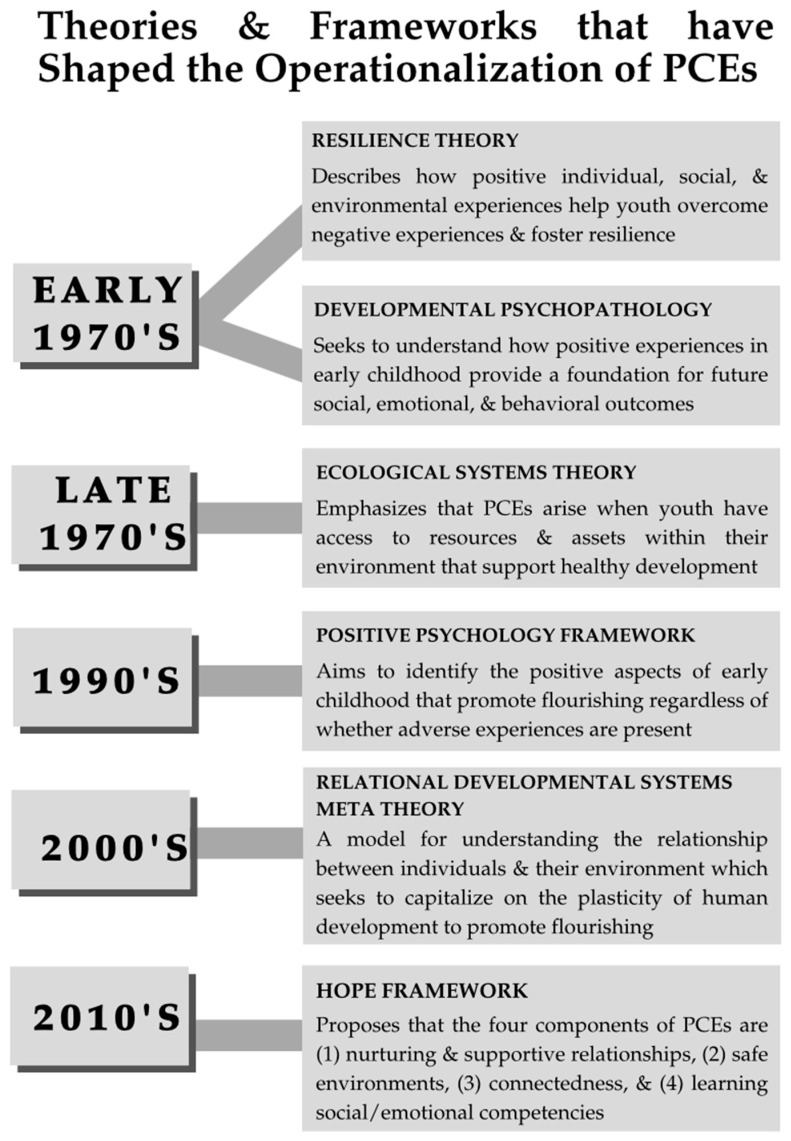
The concept of and working definition for domains of positive childhood experiences (PCEs) have evolved over half a century.

There is no consensus among scholars or clinicians about how to measure PCEs. To resolve this gap, we operationalized PCEs by assessing their promotive and protective effects during early childhood development (see Supplemental Table S1). Scientists across several fields have established various theories, frameworks, and paradigms for understanding how PCEs may help children and youth overcome negative experiences. Each of these frameworks established its own instrument for measuring different aspects of childhood resilience-building skills, mostly designed for retrospective reporting by adolescents and adults. We maintain that PCEs measured during early childhood should inform us of parenting styles, parent–child bonding opportunities, and family dynamics used for building child resilience skills. Treatments may then be designed to overcome the detrimental effects of the Social Drivers of Health, and thereby preserve early childhood development, child health, positive adaptation, and protection throughout the life course (see Figure 1).

Resilience theorists in the 1970s laid the groundwork for defining PCEs by positing that researchers should understand the positive environmental, societal, and individual variables that disrupt maladaptive developmental trajectories (Masten, 2018). Consequently, resilience theorists began to diverge from the dominant deficit-based approach of the time that strongly emphasized the role of ACEs and, instead, turned toward a more strength-based approach. Resilience theorists then explored how PCEs act as protective factors by attempting to understand the unique individual, familial, and community-level factors that can promote resilience in the face of adversity (Shean, 2015). However, even within the resilience theory framework, there is often disagreement over which level(s) and which factor(s) operating at each level are integral to fostering child resilience. Some theorists believe positive family and social relationships to be the most crucial in fostering resilience, while others believe individual factors (e.g., temperament) may help to better understand the variable effects of PCEs (Shean, 2015). In recent years, resilience researchers have begun to describe resilience as a process that adapts over time, and the key positive factors that promote resilience may change over the course of one’s lifespan. For instance, in early childhood, parent–child quality time and close caregiver relationships are seen as crucial for fostering resilience and promoting child flourishing (Southwick et al., 2014; Waters et al., 2023). As an individual ages, however, they may start to draw strength from peers, friendships, role models, spirituality, or other resources available to the individual over time. In any case, resilience theorists across the board seem to agree on understanding how PCEs help youth overcome past traumatic experiences.

The Developmental Psychopathology (DP) framework underscores the importance of PCEs in early childhood, believing that these experiences provide a foundation for the future social, emotional, and behavioral outcomes in adulthood. Developmental psychopathologists have explored how early social experiences such as attachments with caregivers, relationships with peers and teachers, and a positive sense of self can buffer maladaptation in the presence of adversity and contribute to positive functioning in the absence of adversity (Narayan et al., 2018). The DP framework clarifies how PCEs can act as both protective and promotive factors, understanding that the absence of negative experiences alone is not enough to bolster one’s health and well-being. By distinguishing these two aspects of PCEs, the DP framework enhances the operationalized definition of PCEs and captures the complex nature by which PCEs operate over the course of child development.

Around the same time as resilience theorists, Bronfenbrenner (1979) was developing the Ecological Systems Theory of Human Development. The theory examines the complex interactions between an individual child and various social and physical environments, understanding that these relationships between a young individual and their surroundings widely influence their health outcomes. According to the Ecological Systems Theory, PCEs arise when children have access to nested, interactional resources that support positive childhood development (Bronfenbrenner, 1979; Cicchetti & Lynch, 1993). These PCEs can occur in *microsystems*, which include a child’s immediate environment (i.e., among family, school, peers), *exosystems*, which encompass environments that indirectly influence a child (i.e., relationship between parents, caregiver’s place of employment, community resources), and in *macrosystems*, which incorporate larger societal values and political, cultural, or religious structures that influence the society in which the child grows up (Hosek et al., 2008). From this perspective, one begins to understand how cultural variations impact the construction and availability of PCEs. However, little attention was given to capturing these variations across different cultures and contexts. In recent years, researchers have begun to address this knowledge gap (Crouch et al., 2022; Geng et al., 2021; Oge et al., 2020), but much further research is needed to understand how culture influences PCEs.

The Positive Psychology framework builds on the earlier conceptions of PCEs by emphasizing human flourishing as distinct from resilience. Positive psychologists have defined well-being, a measure of human flourishing, in two distinct ways: Firstly, through subjective well-being, defined as positive affect (e.g., happiness) and life satisfaction. Secondly, through psychological well-being, defined as positive relationships, healthy environments, autonomy, and life purpose (Benoit & Gabola, 2021). Despite the paucity of research applying the positive psychology framework to early childhood development, researchers typically emphasize positive social relationships and healthy school environments as crucial to promoting the physical and psychological well-being of children (Baker et al., 2017; Benoit & Gabola, 2021). Unlike resilience theory, the positive psychology framework concerns itself more with how PCEs operate as promotive factors and aims to identify the positive aspects of human experiences and successful adaptation, regardless of whether adversity is present.

To further capture the nuance of defining and operationalizing PCEs, the Relational Developmental Systems (RDS) Paradigm offers an alternative conceptual approach to developmental science. The RDS framework reflects a meta-model for understanding the dynamic relationships between individuals and their environments across generations and throughout the life course (Lerner, 2006). The RDS framework underscores the processes that regulate exchanges between individuals and their contexts (Lerner & Schmid-Callina, 2013). When these “processes” foster mutually beneficial individual contextual relations, they are considered adaptive. Additionally, the RDS paradigm affirms the presence of plasticity in human development, which arises from systematic changes that may occur between an individual and their numerous evolving contexts (Overton, 2013a, 2013b). As a whole, the RDS paradigm puts forth the promising idea that by capitalizing on the plasticity of human development, one can make changes to the individual context relationships that foster favorable developmental trajectories. In other words, the RDS framework provides a theoretical foundation for optimizing human development, particularly during early childhood.

The most recent framework for understanding and operationalizing PCEs is the Health Outcomes from Positive Experiences (HOPE) framework. The HOPE framework emphasizes the promotion of PCEs that foster a strong foundation of positive physical and mental health, along with positive cognitive, social, and emotional outcomes later in life (Guo et al., 2022). Researchers tend to focus on the HOPE framework because it brings together key features of existing frameworks to comprehensively examine PCEs within four central components: (1) nurturing and supportive relationships, (2) safe and protective environments, (3) constructive social engagement and connectedness, and (4) learning social and emotional competencies (R. D. Sege & Harper Browne, 2017). These components combine elements of the resilience theory (e.g., identifying factors that promote resilience), ecological systems theory (e.g., emphasis on environmental interactions), positive psychology (e.g., identifying the core elements that contribute to flourishing), and the DP framework (e.g., the emphasis on early childhood events influencing future outcomes). As such, the HOPE framework provides the clearest, most nuanced definition of PCEs capable of informing measurement tools and intervention strategies. Even so, the HOPE framework does not consider the child’s age and age-based progression of biopsychosocial competency when defining PCEs. We consider this a major reason why PCEs have not been measured in very young children.

## 4. Evolution of the Measurement of PCEs

**Figure 2:**
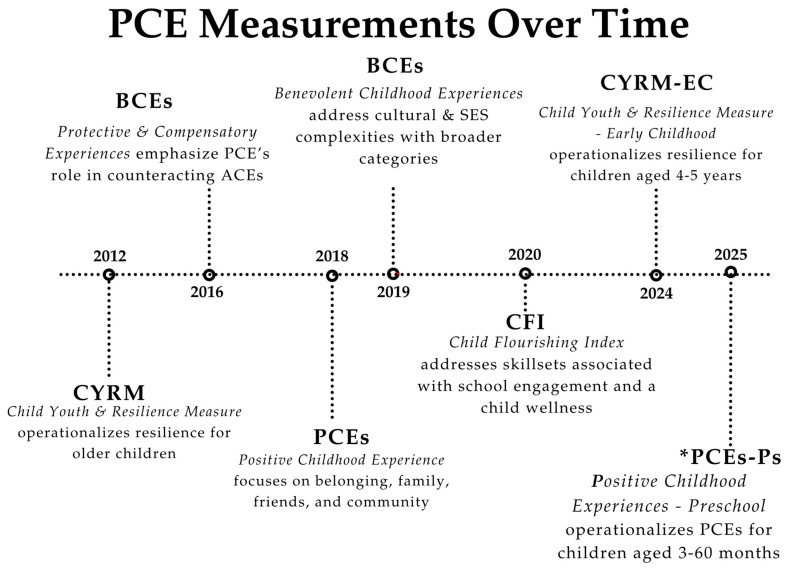
* The Child Wellness Laboratory at Stanford University School of Medicine is prospectively testing a Positive Childhood Experiences—Preschool instrument for use with children between the ages of 3–36 months in a registered clinical trial (NCT07011082). This study aims to enroll a minimum of 450 children with follow-up visits at 6-month intervals for 2 years to examine the exposures to ACEs and PCEs on child developmental and health status.

Existing theories and frameworks have not defined PCEs to be measured prospectively in preverbal children. With a concentration on developing a prospective instrument to capture PCEs during preverbal child development, we summarize the contributions of experts in the field of resilience and well-being (see Table 1) along with the historical timeline for the introduction of PCE measurements (Figure 2).

Early frameworks for understanding parental influence on child development laid the foundation for contemporary measures of PCEs. Instruments like the Parental Investment in Children (PIC) scale and the Parental Bonding Instrument (PBI) were pivotal in assessing parental behaviors and their impact on children’s socioemotional development. The PIC scale evaluates parents’ socioemotional investments, such as time and emotional support, which are critical for fostering child well-being and resilience (Bradley et al., 1997; Whiteside-Mansell et al., 2001). Similarly, the PBI captures dimensions of care and overprotection, offering insights into how parental attitudes shape mental health outcomes in their children (Sato et al., 2021; Suzuki & Kitamura, 2011). These tools reflect an early shift towards recognizing the promotive role of parental behaviors and predating the principles of positive psychology.

Inspired by these efforts, “40 Developmental Assets” (Search Institute, 1999) and the Child Youth and Resilience Measure (CYRM) used at ages 12–23 years (Liebenberg et al., 2012; Ungar & Liebenberg, 2011) and the CYRM-Early Childhood (Lipscomb et al., 2025) used with children aged 4–5 years were developed to characterize the factors associated with resilience. By surveying children and young adults in various age groups, these measures aimed to assess internal factors (such as social-emotional strengths, values, and commitments) and external factors (such as family support and community empowerment) that facilitate developing resilience. Although the CYRM conducted research in 11 countries (Ungar & Liebenberg, 2011), it is unclear if the nuances of language and intent were maintained cross-culturally. Validation studies were primarily performed in English-speaking countries or were reduced and adjusted to address specific needs.

Later psychological research found that fewer than 50% studies validated the results from the original CYRM study (Open Science Collaboration, 2015). The CYRM identifies factors such as religious affiliation or cultural awareness, as well as patterns of intergenerational disparities, to characterize risk factors in adolescent populations (Artuch-Garde et al., 2022; Sanders et al., 2017) and also exemplifies the difficulties of conducting validation studies with varied populations.

With the introduction of the Protective and Compensatory Experiences (PACEs) scale in 2016, the role of PCEs was directly emphasized in mitigating the untoward effects of ACEs (Morris et al., 2016). While PACEs began as a retrospective measure to examine an adult’s recollection of their childhood, it is now seen as a tool for parents to help their children handle stress. With categories such as “parent/caregiver unconditional love” and “having a hobby”, PACES encourages parents to give constructive suggestions and directly provide emotional guidance. In essence, PACES is a reactive measure that allows parents to intervene once they identify the impact of ACEs, such as stress (Morris & Hays-Grudo, 2023).

This scale eventually led the way to the PCEs scale (C. Bethell et al., 2019) and the Benevolent Childhood Experiences (BCEs) scale (Merrick et al., 2019), two validated retrospective measures that highlight the importance of cumulative PCEs recalled during adulthood. The PCEs scale focused more on relationships rather than the child’s daily activities; total scores were calculated using the frequency of positive responses, such as “always” or “almost always,” and findings supported the mental health benefits of PCEs for adults (C. Bethell et al., 2019). The BCEs scale was developed to address cultural complexities as well as differences in region and socioeconomic status and was intended primarily for adults with a history of child maltreatment, among other high-risk participants, and continues to be validated in international settings (Narayan et al., 2018). In studies using either method, researchers often compare the number of PCEs or BCEs against the ACEs (Crandall et al., 2020, 2019; Han et al., 2023; Narayan et al., 2018).

To account for cultural or socioeconomic differences, the BCEs focus less on specific activities and include a broader category of factors. In terms of the cultural context, the PCEs scale equates childhood support with the ability to discuss feelings with family, while the BCEs acknowledge cultural differences that may not normalize this behavior even in healthy children. Consequently, the BCEs include broader questions about comforting beliefs, not presuming family discussion as the sole method of overcoming adversity. The same applies to differences in socioeconomic status or geographic diversity. Whereas the PACEs scale highlights healthy behaviors such as outdoor activities and hobbies, the BCEs scale includes a more general statement of whether participants had opportunities to have a good time. This ensures that a family’s ability to support specific activities does not skew assessments of a child’s positive experiences, thus preventing cultural and socioeconomic factors from confounding the results (Almeida et al., 2021; Hou et al., 2022; Narayan et al., 2018). With adult participants, PACEs, PCEs, and BCEs used one primary list of questions without incorporating the nuance of developmental changes. All scales require responses based on one’s collective childhood experiences.

Bethell utilized data from the National Survey of Children’s Health to develop the Child Flourishing Index (CFI), focusing on the child’s willingness to learn, complete tasks, and maintain emotional regulation in challenging situations (C. Bethell et al., 2019). The CFI gained prominence with the CAP-2030 initiative in 2020, spearheaded by the WHO-UNICEF-*Lancet* Commission to promote child well-being (Clark et al., 2020). The CFI was used in conjunction with health, education, and nutrition metrics to rank countries based on the prevalence of “flourishing” children in comparison to other sustainability goals and general wealth (Clark et al., 2020). This scale was validated based on its association with school engagement, parental encouragement of flourishing behaviors, and parent–child connection in the context of family resilience. Unlike other scales that focus on environmental factors affecting a child’s development, the CFI prioritizes tangible, direct outcomes that communities and schools can address. For instance, it assesses a child’s ability to manage their emotions, a skill that can be nurtured in many environments (schools, extracurricular and cultural activities in the community, etc.). Thus, this index encourages family and community involvement in addressing the child’s developmental needs (C. D. Bethell et al., 2019).

This section does not include a comprehensive list of measures and tests developed to measure PCEs or other factors promoting child development. Given the complexities of validating and adapting measures across populations, we only selected measures with multiple validating studies that can inform future research in this field.

## 5. Discussion

This narrative review underscores the need for an instrument to prospectively measure PCEs in preverbal children or other nonverbal populations increasingly susceptible to ACEs or other forms of adversity. Positive and negative experiences in early childhood particularly play a critical role in shaping future health outcomes, often mediated or even moderated by the social drivers of health (SDH). A validated inventory for preschool PCEs lags behind established measures available for ACEs (i.e., Pediatric Adverse Childhood Experiences and Related Life Events Screener, PEARLS 2.0) (Ye et al., 2023) and SDH factors like economic stability, quality education, healthcare access, safe environments, and social networks (Freedland et al., 2025; Jawad et al., 2025; Peddu et al., 2025). These factors not only influence the prevalence and impact of ACEs but also undermine access to PCEs, which are essential for fostering resilience, emotional regulation, and long-term flourishing, perpetuating cycles of adversity and health inequities (Anand et al., 2019; CDC, 2024a). Addressing these inequities requires systemic interventions that prioritize equitable access to resources for PCEs, such as community-based programs, policy reforms, and culturally sensitive support systems. Future research on targeted interventions to mitigate the long-term effects of ACEs with or without SDH disparities, to ultimately improve the developmental trajectories for children in vulnerable populations, essentially requires objective and validated measures for ACEs, PCEs, and SDH. At present, however, an objective measure for prospective evaluation of PCEs has not been devised or validated.

Moreover, within the intricate tapestry of early childhood, the concept of PCEs emerges as a pivotal determinant of both current and future well-being and resilience. The current state of PCE research highlights the challenges of defining, operationalizing, and measuring PCEs. Researchers across various fields have struggled to come to a consensus on defining PCEs, as evinced by the differing frameworks and scales reviewed above. This challenge is further complicated by the lack of evidence describing the exact mechanisms by which PCEs influence the developing brain and behaviors in early childhood. Though positive and negative childhood experiences impact physical and mental health outcomes in adulthood, researchers have yet to define how PCEs influence resilience-building in early childhood, and how this relationship contributes to brain and behavioral development. Inconsistent methods utilized in studies examining the neurological underpinnings of PCEs limit our understanding of how PCEs mitigate the adverse effects of ACEs and promote wellbeing even in the absence of adversity (Zhang et al., 2023). Furthermore, most neurobiological studies on PCEs have solely focused on child-caregiver relationships. As such, future research must examine a greater scope of PCEs using consistent methodologies to distinguish their roles in fostering well-being, resilience, and flourishing.

While attempting to operationalize PCEs, each framework and theory discussed in this review provides a unique perspective for examining PCEs. Yet, each field seems to touch on how positive experiences demonstrate the presence of resources and assets that promote the ideal conditions for healthy childhood development. Consequently, A mono-cultural bias toward Western ideals (e.g., individual autonomy and verbal emotional disclosure) can limit the generalizability of PCE frameworks. Cross-cultural research shows that culturally normative PCEs differ in expression and valence: collectivist contexts often prioritize family rituals, filial obligations, and intergenerational support as normative developmental assets, whereas individualist contexts emphasize autonomy and self-expression (Markus & Kitayama, 1991; Chao, 1994). Measurement development therefore requires not only linguistic translation but also conceptual equivalence. Acculturation adds complexity because first- and second-generation families may hold hybrid practices, and the salience of specific PCEs can shift across the acculturation gradient; acculturation stress itself can interact with PCEs to alter their promotive or protective effects. We recommend that future instrument development include culturally diverse advisory panels, cognitive interviews across target communities, and explicit tests of measurement invariance across cultural and acculturation strata (Markus & Kitayama, 1991; Chao, 1994; Narayan et al., 2023).

## 6. Conclusions

The measurement of PCEs highlights several limitations in the current literature. Firstly, prior measures of PCEs, including the PCEs and BCEs scales, are dependent on an adult’s recollection of their childhood, which introduces recollection bias and bias by indication. Responses to questions about the past may be influenced by an individual’s current feelings about their past childhood experiences rather than what they actually experienced. Secondly, there are also concerns regarding a lack of specificity. While the PCEs scale somewhat incorporates frequency, the BCEs scale is based on binary assessments. Additionally, changes in the intensity and cumulative frequency of PCEs during childhood are often not captured in the current measures. Thirdly, there is a lack of standardization of these measures across various ethnic, cultural, and socioeconomic backgrounds, thus limiting the generalizability of PCE research. While the BCE and CYRM scales begin to capture some differences, variability in the quality and strength of these cross-cultural studies points to a need to prioritize consistent and reliable cross-cultural validation.

## Figures and Tables

**Table 1:** PCE Measures by Age.

Age Range	Positive Childhood Experiences (PCEs) Scale ^1^	Adult Attachment Questionnaire ^2^	Beneficent Childhood Experiences (BCEs) Scale ^3^	Child Flourishing Index (CFI) ^4^
0–2 years	Feeling safe and protected by an adult at home	Formation of primary attachment bonds	Safe, responsive caregiving	Child affectionateness and tenderness
3–5 years	Able to talk with family about feelings	Development of internal working models of relationships	Positive sense of self	Early signs of competence and resilience
6–11 years	Having at least two non-parent adults who genuinely care	Expansion of attachment network to include peers and other adults	Supportive peer relationships	Curiosity and purpose
12–18 years	Feeling of belonging in school Feeling supported by friends Enjoyment in participation in community traditions	Refinement of attachment styles Formation of romantic attachments	Community involvement Positive school experiences	Meaningful relationships Sense of personal growth
18+	Retrospective evaluation of childhood experiences	Ease in becoming emotionally close to others Comfort level without close emotional relationships Ease in having dependents	Positive childhood memories Supportive family relationships Sense of community belonging	Life satisfaction Purpose and meaning Character and virtue Social relationships Financial stability

Note. Each scale was devised by the following researchers/groups, respectively: ^1^ Dr. C. Bethell et al. (2019). ^2^ (Collins & Read, 1990) building upon and as cited in Hazen and Shaver (1987); ^3^ Dr. Narayan et al. (2018); ^4^ Clark et al. (2020).

## Data Availability

No new data were created or analyzed in this study. Data sharing is not applicable to this article.

## Supplementary Materials

The following supporting information can be downloaded at: https://www.mdpi.com/article/10.3390/bs15111432/s1, Table S1: Positive Childhood Experiences—Preschool (PCE-P).

## Abbreviations

The following abbreviations are used in this manuscript:

ACEs	Adverse Childhood Experiences
BCES	Benevolent Childhood Experiences
CDC	Centers for Disease Control and Prevention
CFI	Child Flourishing Index
CYRM	Child Youth & Resilience Measure
CYRM-EC	Child Youth & Resilience Measure-Early Childhood
dACC	dorsal anterior cingulate cortex
DP	Developmental Psychopathology
HOPE	Health Outcomes from Positive Experiences
PACES	Protective and Compensatory Experiences
PBI	Parental Bonding Instrument
PCEs	Positive Childhood Experiences
PCES-P	Positive Childhood Experiences—Preschool
PIC	Parental Investment in Children
RDS	Relational Developmental Systems
rsFC	resting-state functional connectivity
SAI	Socioeconomic Adversity Index
SDH	Social Driver of Health
SDoH	Social Determinants of Health
SES	Socioeconomic Status
