# Supplementary material for: Promoting Child Wellness: A Narrative Review of Positive Childhood Experiences
Source: Behav Sci (Basel). 2025 Oct 22;15(11):1432. doi: 10.3390/bs15111432 (PMC12649361; doi:10.3390/bs15111432)
Supplement: Supplementary file 1 [file behavsci-15-01432-s001.zip › behavsci-3815212-supplementary.pdf]

Supplemental Table S1

Enrolled Child's Name: ______________________________

Enrolled Child's Study ID: ___________________________

Stanford Child Wellness Lab

©Copyright 2023 All rights reserved

POSITIVE CHILDHOOD EXPERIENCES (Adolescent/Adult)

(From: ***"Raising a Healthy Child"*** Dr. Meena Chintapalli, Prowess Publishing, ©2020, ISBN: 978-1-5457-4721-6 ***"Childhood Matters"*** Sudha Kudva (Ed. Lorien Holland), Percetakan Kencana, ©2016, 2nd Edition 2017; ISBN: 978-967-14523-1-8).

Age 3-9 months

I make eye contact and talk softly to my baby….	**Almost never 0%-10%**	**Rarely 10%-30%**	**Sometimes 30%-50%**	**Frequently 50%-70%**	**Almost always 70%-90%**
- while feeding or breastfeeding?					
- during diaper change & bathing?					
- when the baby is awake & alert?					
I pick up and soothe my crying baby					
Give baby massage or touch therapy					
Offer baby tummy time + soft music					
..picture books, interactive play, toys					

Age 9-18 months

I help my infant to….	**Almost never 0%-10%**	**Rarely 10%-30%**	**Sometimes 30%-50%**	**Frequently 50%-70%**	**Almost always 70%-90%**
- crawl, cruise, walk, run when ready					
- reduce stranger/separation anxiety					
- explore new objects, spaces, books					
- learn new words, feeding, dressing					
- have regular routines, sleep times					
- play, read, sing with peers/parents					

Age 18-36 months

I help my toddler to…..	**Almost never 0%-10%**	**Rarely 10%-30%**	**Sometimes 30%-50%**	**Frequently 50%-70%**	**Almost always 70%-90%**
- self-regulate emotions & behaviors					
- express theirs needs & experiences					
- grow self-confidence, self-identity					
- make positive & healthy choices					
- learn new words, skills, & games					
- solve new problems and puzzles					

Age 36-60 months

I help my preschooler to…..	**Almost never 0%-10%**	**Rarely 10%-30%**	**Sometimes 30%-50%**	**Frequently 50%-70%**	**Almost always 70%-90%**
- gain mastery over bodily functions					
- learn to dance, sing, draw, & read					
- gain autonomy, earn rewards/praise					
- develop love, respect, and empathy					
- enhance memory, natural curiosity					
- develop good eating habits and diet					
- practice sleep hygiene, daily routine					
